# A Futures Framework for Clinical AI Governance: Anticipating Emerging Risks, Shifting Roles, and Regulatory Challenges

**DOI:** 10.2196/96152

**Published:** 2026-06-29

**Authors:** Yi Yang, Jialin Liu, Siru Liu

**Affiliations:** 1 Information Center West China Hospital of Sichuan University Chengdu, Sichuan China; 2 Department of Otolaryngology-Head and Neck Surgery West China Hospital of Sichuan University Chengdu, Sichuan China; 3 Department of Medical Informatics West China Medical School of Sichuan University Chengdu, Sichuan China; 4 Department of Biomedical Informatics Vanderbilt University Medical Center Nashville, TN United States

**Keywords:** clinical AI, artificial intelligence, clinical AI governance, futures studies, strategic foresight, health regulation, clinical workforce, emerging risks, digital health policy, artificial intelligence governance

## Abstract

This viewpoint develops the futures framework for clinical artificial intelligence governance (FF-CAIG), a conceptual and anticipatory framework for organizing emerging governance challenges in clinical artificial intelligence (AI). Although life cycle–oriented oversight is increasingly reflected in clinical AI regulation and institutional governance, existing approaches remain more developed for near-term validation, current-state assurance, and retrospective risk detection than for longer-horizon sociotechnical change. This gap is increasingly relevant, as AI systems become more complex, adaptive, and autonomous, and as they become more deeply embedded in care relationships and accountability structures. FF-CAIG is grounded in 3 futures methodologies: the 3 horizons model, scenario planning, and causal layered analysis. It is operationalized through an emerging clinical AI risk taxonomy that links these methods to governance domains. Its practical outputs include horizon classification, risk-domain mapping, scenario stress-testing findings, accountability-chain mapping, and horizon-scaled minimum governance actions for deployment or continued use. Applied across near-term, transitional, and longer-term horizons, the framework proposes cross-horizon priorities, including stronger predeployment equity evaluation, clearer life cycle accountability, clinician AI oversight competencies, and safeguards for increasingly autonomous or AI-mediated care systems. We illustrate FF-CAIG through 3 representative clinical AI deployment patterns and discuss its limitations, including differential compliance burdens, risks of overdocumentation, variable feasibility across jurisdictions, and the need for empirical validation. FF-CAIG is intended not as a prescriptive policy instrument or validated assessment tool, but as a structured analytic approach for regulators, health system leaders, developers, and researchers seeking prospective and systems-oriented approaches to clinical AI governance.

## Introduction

Clinical artificial intelligence (AI) has moved beyond controlled validation settings into routine deployment, supporting risk stratification, documentation, patient communication, and emerging decision support across care settings [1-3]. Regulatory oversight of imaging algorithms, predictive models, and other AI-enabled medical products has also expanded through evolving US Food and Drug Administration (FDA) and European Union frameworks [4,5]. Although clinical AI governance increasingly incorporates life cycle–oriented elements, including postmarket monitoring and change-management expectations, much oversight remains more mature for near-term assurance and recognized risk signals than for longer-horizon sociotechnical change. Existing approaches, including institutional ethics oversight, regulatory approval pathways, and industry guidance, still focus primarily on risks such as bias, privacy breaches, performance degradation, and safety events that can be specified, monitored, or retrospectively detected [4-7].

This orientation is increasingly challenged by the iterative, updateable, and cross-functional nature of advanced clinical AI [8]. Recent regulatory developments, including the FDA’s life cycle–focused guidance for AI-enabled device software functions and the EU AI Act’s postmarket obligations for high-risk systems, indicate that clinical AI governance is no longer limited to static premarket approval [4,7,9]. The remaining gap is more specific: current life cycle–oriented approaches are better developed for planned or bounded change, defined postmarket obligations, and measurable performance signals than for evolving clinical functions, foundation model–based tools, distributed accountability, and relational effects that may shift across contexts and over time. In health care, this challenge is especially consequential because safety, accountability, and trust depend not only on model performance but also on role clarity, implementation quality, and institutional capacity to detect and respond to change [1,3].

The governance challenge posed by clinical AI is therefore not only technical but also temporal. Although regulators and health systems increasingly recognize the need for life cycle oversight, existing mechanisms still depend substantially on foreseeable changes, measurable postdeployment signals, and recognized failure modes. Clinical AI is evolving in ways that may generate governance problems before they are fully captured by conventional postdeployment oversight [10,11]. Some risks may emerge gradually, including clinician overreliance, rising verification burden, workflow dependence, and fragmentation of governance across institutions and jurisdictions [12-15]. Generative AI systems used for longitudinal communication, coordination, or triage may also reshape patient-clinician relationships in ways that existing frameworks for consent, accountability, and duty of care do not fully anticipate [16,17]. This concern is increasingly recognized in international guidance, including recent World Health Organization work on large multimodal models in health [18].

Futures studies provide a systematic but underused set of approaches for anticipating long-term change and emerging uncertainty [19,20]. Although organizations such as the Organisation for Economic Co-operation and Development and World Health Organization have identified horizon scanning and scenario analysis as valuable governance tools under conditions of rapid change [21,22], these methods have not been systematically integrated into clinical AI governance. As a result, longer-horizon concerns, including systemic dependency, workforce disruption, and regulatory obsolescence, often remain secondary to current-state compliance and near-term risk mitigation [15,23,24]. Existing frameworks provide important foundations, but they offer limited operational guidance for anticipating risks that may emerge beyond conventional life cycle monitoring, compliance assessment, or management-system processes. The futures framework for clinical artificial intelligence governance (FF-CAIG) addresses this gap by integrating futures methods with clinical AI governance.

In this viewpoint, we introduce FF-CAIG, grounded in core futures methodologies [19,20] and centered on patient safety and health equity as foundational principles [18,25]. The framework addresses 3 interconnected domains: emerging systemic and clinical risks, evolving stakeholder roles across the care ecosystem, and regulatory responses that can better keep pace with technological change. FF-CAIG is designed to be prospective, adaptive, and applicable across organizational governance, regulatory policy, and clinical practice. FF-CAIG is intended for clinical AI systems whose deployment may create clinically, organizationally, ethically, or relationally consequential governance challenges. Its scope includes high-risk clinical decision-support systems, foundation model–based clinical tools, patient-facing communication or triage systems, and administrative or documentation tools that meet this threshold. It is not intended for low-impact administrative automation with no plausible effect on clinical decisions, patient-facing communication, safety, equity, or professional accountability; governance intensity should scale with autonomy, clinical consequences, deployment context, and horizon classification. We argue that it offers both a conceptual contribution to health informatics scholarship and a practical tool for regulators, health system leaders, professional bodies, and clinical AI developers seeking more anticipatory clinical AI governance under technological uncertainty.

**Figure 1:**
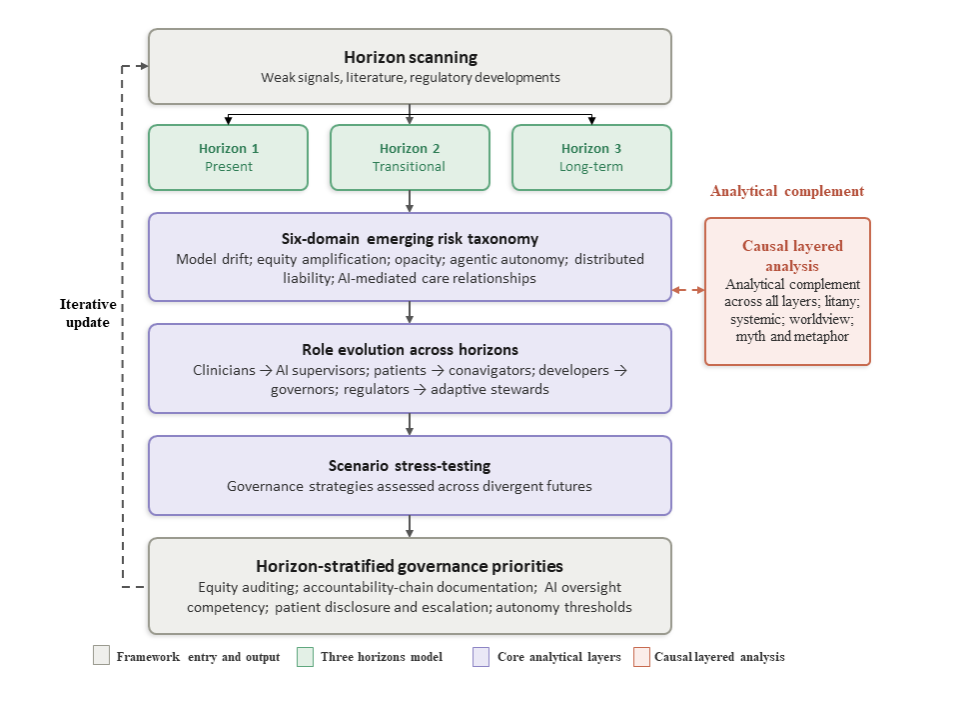
FF-CAIG: integrated analytical cycle for clinical AI governance. AI: artificial intelligence; FF-CAIG: futures framework for clinical artificial intelligence governance.

FF-CAIG is not intended as a simple aggregation of established foresight methods. Rather, it is designed as an integrated governance framework in which each component addresses a distinct limitation of oversight models that primarily emphasize current-state performance, compliance, and post hoc risk management in clinical AI [10,15]. These 3 methods were selected because they address complementary needs in anticipatory clinical AI governance. The three horizons (3H) model supports temporal classification of risks across near-term, transitional, and longer-term phases; scenario planning stress-tests governance priorities under regulatory and institutional uncertainty; and causal layered analysis (CLA) examines the structural assumptions, professional role expectations, institutional incentives, and cultural narratives that shape which risks are recognized and how they are governed [19,23]. Other approaches remain useful for adjacent governance functions: horizon scanning can support evidence gathering, Delphi consensus can support expert agreement, technology road mapping can support implementation planning, risk matrices can support risk scoring, and safety-case methods can support assurance documentation. However, no single method by itself connects temporal transition, policy robustness, and deeper sociotechnical analysis in a way that directly supports clinical AI governance. FF-CAIG integrates these functions into a single analytic framework (Figure 1).

## The Limitations of Present-Oriented Clinical AI Governance

### Overview

Current approaches to clinical AI governance are increasingly incorporating life cycle–oriented elements, including postmarket monitoring, change-control planning, and ongoing obligations for high-risk AI systems [3,4,7,9]. These developments are important and should not be characterized as purely static or reactive. However, most frameworks remain more developed for current-state evaluation, foreseeable updates, and retrospective risk detection than for anticipating longer-horizon changes in clinical roles, institutional accountability, patient relationships, and regulatory adaptation [10,15]. This creates a growing mismatch with the dynamic, distributed, and sociotechnical character of clinical AI in practice.

### Static Approval Models for Dynamic Systems

A central limitation of existing governance frameworks is their reliance on approval models developed for relatively stable technologies, structured around defined intended use, bounded functionality, and evaluation at discrete time points [10,11]. Although this remains useful for narrow and well-characterized applications, it is increasingly strained when applied to updateable, adaptive, or implementation-sensitive AI systems [1,8]. The FDA’s predetermined change control plan framework is an important advance because it recognizes that AI-enabled device software may change after authorization [9]. However, it remains primarily oriented toward planned and bounded change, making it less well suited to emergent behavioral shifts in foundation model–based systems or layered AI ecosystems shaped by model revision and local implementation [24]. In parallel, the European Union’s AI governance architecture introduces conformity assessment and postmarket obligations for high-risk systems [4,7], but practical questions remain about defining material change, monitoring continuously updated systems, and allocating ongoing oversight responsibility.

### Postmarket Surveillance Gaps

A further limitation concerns how postdeployment harms are detected. Conventional postmarket surveillance was designed around discrete, reportable adverse events; yet, the performance of clinical AI may erode gradually as data, populations, and workflows shift after deployment [26,27]. Episodic, incident-triggered reporting is therefore poorly suited to detecting progressive deterioration, which may persist for an extended period before surfacing as an identifiable safety signal [8,10,12]. As clinical AI becomes more embedded in routine care, governance will need to move beyond incident reporting toward continuous, context-sensitive, and performance-aware monitoring [9].

### Accountability Fragmentation

Clinical AI systems are increasingly developed, adapted, integrated, and configured by multiple actors across the system life cycle [1,8,28]. This distributed architecture can make accountability difficult to locate within conventional governance and liability models, particularly when harm arises from interactions among model design, local implementation, workflow integration, and clinical use [15-17]. This limitation creates the need for more explicit accountability allocation, which is addressed later as a core risk domain within FF-CAIG.

### Equity as a Core Governance Requirement

Algorithmic bias is among the most widely recognized risks in clinical AI [29,30]; yet, equity is still often treated as secondary rather than as a core governance requirement [18,25]. Existing frameworks rarely provide operational standards for subgroup performance reporting, proxy variable auditing, or postdeployment equity surveillance [31]. The mechanisms and life cycle requirements of equity-oriented governance are examined later as a core risk domain within FF-CAIG; at this stage, the key point is that the absence of such standards leaves a structural gap that conventional oversight is poorly positioned to close. Taken together, these limitations expose a deeper mismatch between conventional oversight and the temporal, relational, and system-level nature of contemporary clinical AI.

## A Futures Methodology for Clinical AI Governance: The 3H Model

### Overview

Among futures methods, the 3H model offers a useful way to analyze governance during system transition [23]. Instead of treating change as a simple linear progression, it frames transformation as unfolding across 3 overlapping horizons. Horizon 1 (H1) is the currently dominant system, still operationally effective but increasingly marked by structural limitations. Horizon 2 (H2) is the transitional space in which emerging innovations, hybrid arrangements, and competing institutional logics begin to challenge H1 assumptions. Horizon 3 (H3) refers to longer-term system configurations whose values, structures, and practices may initially appear peripheral but can ultimately redefine the dominant order.

### Relevance to Clinical AI Governance

Applied to clinical AI governance, the 3H model offers several advantages over linear forecasting. It recognizes that current governance structures will remain important even as their limitations become more visible, creates analytical space for examining transitional and longer-horizon challenges before they are fully realized [21,22], and links technological change to shifts in professional roles, accountability structures, and care relationships [15,19]. In this sense, the 3H model is not simply a way of organizing time but a framework for understanding how governance logics evolve during sociotechnical transition. This perspective is especially relevant in clinical AI. Recent developments, including the FDA’s predetermined change control plan guidance [9] and the EU AI Act’s postmarket obligations [4,7], represent important advances, but they do not eliminate the need for anticipatory governance [10,24]. As clinical AI moves from relatively bounded tools toward more complex, layered, and adaptive ecosystems, governance assumptions may be challenged differently across successive horizons [32]. Table 1 applies the 3H model to clinical AI governance by organizing plausible conditions across near-term, transitional, and longer-term horizons.

**Table 1:** Three horizons model applied to clinical artificial intelligence (AI) governance.

Domain	Horizon 1: present system	Horizon 2: transitional emergence	Horizon 3: longer-term reconfiguration
Temporal orientation	Near term	Transitional period	Longer term
Dominant paradigm	Point-of-care AI tools, human-in-the-loop support, device-centered regulation	Increasing task delegation, workflow-embedded foundation-model capabilities, and iterative system updates	More autonomous, AI-mediated care infrastructures and cross-pathway coordination
Primary governance challenge	Premarket validation, transparency, bias and performance assessment, and fit within existing regulatory pathways	Oversight of iteratively updated systems, layered accountability, workflow dependence, and role transition	Responsibility, consent, disclosure, and professional accountability in low-human-intervention or AI-mediated care

### Governance Implications of the 3H Model

Governance investments focused only on H1 realities, such as device classification, one-time approval logic, and narrow predeployment evaluation, may be insufficient for H2 conditions characterized by iterative updating, layered accountability, and partial delegation [10,15]. They are even less suited to H3 settings in which clinical AI functions less as a bounded tool and more as a persistent mediating infrastructure across episodes of care [32]. Governance should therefore be designed across all 3 horizons simultaneously, even when later horizons remain only partially formed [19,20]. The 3H model thus reframes clinical AI governance as a problem of system transition rather than isolated product oversight. It helps regulators, health systems, and professional bodies identify where current mechanisms remain adequate, where hybrid approaches are needed, and where new forms of oversight may become necessary [19,21,22]. The goal is not speculative prediction but anticipatory preparedness.

## An Emerging Risk Taxonomy for Clinical AI Governance

### Overview

Drawing on a targeted narrative review and literature-informed conceptual synthesis of clinical AI governance literature, regulatory and policy documents, and analogous high-stakes AI domains, we identify 6 risk domains that warrant anticipatory attention in clinical AI governance (Table 2) [8,15,18,31]. Because this paper is a viewpoint, FF-CAIG was developed through a structured synthesis rather than a preregistered protocol, formal expert consensus process, or empirical coding study. The synthesis involved targeted source review, identification of recurring governance concerns, iterative grouping into risk domains, mapping to futures methods, and assessment of links to practical governance outputs. Sources were prioritized when they addressed risks relevant to clinical decision-making, patient safety, institutional accountability, professional responsibility, equity, trust, or governance design, not technical performance alone. Recurring concerns were iteratively compared and grouped according to their dominant governance gap: technical reliability, structural accountability, or relational quality. Domains were retained when they were recurrent across sources, relevant to multiple stakeholders, applicable across different clinical AI deployment patterns, and linked to actionable governance responses. The taxonomy is intended to support anticipatory governance by distinguishing risks that are already visible, emerging, or likely to become more consequential over time. It is therefore presented as a viewpoint-based analytic framework, not as a systematic review–derived, empirically coded, or exhaustive classification.

In this taxonomy, H1 denotes risks already observable in current clinical AI deployments; H2 denotes risks that become more consequential, as systems become iteratively updated, workflow-embedded, foundation model–based, or partially delegated; and H3 denotes longer-term risks associated with more autonomous, persistent, or AI-mediated care infrastructures.

**Table 2:** Emerging risk taxonomy and horizon placement for clinical artificial intelligence (AI) governance.

Risk domain	Illustrative manifestation	Horizon (H)	Governance gap	Rationale for horizon placement
Model drift and silent failure	Model performance declines as populations, data pipelines, or workflows change	H1-H2	Inconsistent postmarket surveillance and drift response	Already observable in current systems (H1); harder to detect, as systems become iterative and workflow-embedded (H2)
Algorithmic equity amplification	Nonrepresentative data lead to poorer performance for underserved groups	H1-H3	Limited equity auditing and subgroup performance standards	Documented in current systems (H1); may scale with foundation models (H2); may become structurally embedded without life cycle equity governance (H3)
Opacity and explainability	Clinicians cannot explain AI recommendations or justify reliance on or overrides of AI outputs	H1-H3	Weak explainability, disclosure, and consent expectations	Present in current tools (H1); intensified by foundation models (H2); more consequential, as AI mediates longitudinal care (H3)
Agentic autonomy	AI performs multistep tasks, such as triage or protocol execution, with limited real-time human review	H2-H3	Unclear oversight standards, autonomy thresholds, and escalation rules	Primarily an emerging risk beyond most current human-supervised deployments; becomes more salient as delegation increases (H2-H3)
Distributed liability	Harm arises across developers, integrators, deployers, and clinicians without clear responsibility	H1-H3	Poor accountability allocation across sociotechnical systems	Relatively clearer for bounded tools (H1); harder to assign, as multiactor and longitudinal workflows expand (H2-H3)
AI-mediated care relationships	Longitudinal AI tools reshape communication, trust, and continuity of care	H2-H3	Limited standards for relational quality, continuity, and access to human review	Early forms are emerging in patient-facing tools (H2); consequences increase when AI persistently mediates care (H3)

### Model Drift and Silent Failure

As noted earlier, clinical AI may become less reliable after deployment, and this deterioration often develops gradually rather than as a discrete failure event. The result is the risk of silent failure: a system continues to operate within its nominal range while its clinical usefulness declines and the problem remains undetected [12,26,27]. Existing governance frameworks remain inconsistent in how they define drift, monitor performance change, or trigger remedial action [8,10]. A prospective governance approach should therefore specify minimum standards for drift detection, reporting cadence, cohort-disaggregated monitoring, and response protocols that activate when clinically meaningful deterioration is detected [9].

### Algorithmic Equity Amplification

The risk that clinical AI may amplify existing inequities is well established [29,30]. These inequities may arise through underrepresentation of historically marginalized groups in training data, proxy variables that encode racial or socioeconomic disadvantage [30], and optimization targets that are misaligned with equity goals. They may also be reinforced by deployment contexts that concentrate advanced tools in already well-resourced institutions [31]. As more general-purpose and foundation model–based systems are deployed across heterogeneous populations, these mechanisms may operate at greater scale and become harder to attribute [33,34]. A futures-oriented governance approach should therefore move beyond documenting disparities after deployment and instead define minimum equity-governance requirements across the system life cycle.

At minimum, high-impact clinical AI systems should undergo subgroup-disaggregated evaluation before deployment, periodic postdeployment reassessment after major updates or material workflow changes, and formal review and remediation planning when subgroup performance falls below prespecified, context-specific clinical acceptability thresholds [9,18,29,31]. These thresholds should be defined prospectively according to the system’s intended clinical function, risk level, and potential consequences of error and may include minimum subgroup performance levels, calibration requirements, maximum allowable subgroup performance differences, and minimum sample-size or confidence requirements for reliable subgroup estimates. Governance should also specify which clinically relevant and equity-relevant subgroup variables are monitored, who is responsible for reviewing disparity signals, and what remedial actions are triggered when inequitable performance is detected. Remediation pathways should be proportionate to the severity and persistence of the disparity and may include governance review, root-cause analysis, enhanced human review, temporary restriction of use, recalibration, retraining, workflow redesign, revalidation, or suspension when safety or equity criteria are breached. In this framework, equity is not only an ethical principle but also an auditable governance function [18,25,31].

### Opacity and Explainability

Foundation model–based and other complex clinical AI systems pose governance challenges that differ from those of more narrowly bounded tools because they often have broader capabilities, more variable downstream uses, and behaviors that are harder to characterize before deployment [34,35]. Under these conditions, clinicians may be unable to explain AI recommendations to patients, justify overrides in the medical record, or determine whether a model’s output reflects clinically relevant reasoning or spurious correlations [10,24]. This creates governance pressure not only around technical explainability but also around disclosure, informed consent, documentation expectations, and professional accountability. Governance frameworks should therefore define minimum expectations for explainability proportional to clinical risk, including requirements for user-facing disclosure, justification of override or reliance in high-impact settings, and documentation standards for AI-supported decision-making [10,34,35].

### Agentic Autonomy

Increasingly, agentic clinical AI presents a major governance challenge on the medium- to longer-term horizon. These systems may support multistep activities such as triage, care coordination, chronic disease management, or protocolized follow-up [2,28]. Although current deployments remain substantially human-supervised, ongoing development trajectories suggest that broader action spaces and more consequential forms of delegated decision support are likely to become more salient over the medium- to longer-term horizon [32]. Existing governance frameworks do not clearly define how human oversight should scale with autonomy, what level of review remains meaningful, or how accountability should be allocated when AI-supported action contributes to patient harm [8,17]. Governance should therefore move beyond a simple human-in-the-loop requirement toward clearer standards for autonomy thresholds, escalation rules, and minimum conditions for meaningful human control [36].

### Distributed Liability in Multicomponent Systems

Accountability in clinical AI is difficult not only because some systems are opaque but also because responsibility is distributed across the development, integration, deployment, and use pathway [1,8]. Patient harm associated with AI-supported care may arise from model development, platform integration, local implementation, and downstream clinical oversight, without any single actor bearing sole responsibility [17]. Conventional medical liability frameworks are poorly suited to this layered architecture. Accountability should therefore be addressed prospectively, with responsibilities specified before deployment and not only after harm occurs [15,17]. One practical approach is to require accountability-chain documentation as a condition of deployment authorization [8,15,37]. This documentation should identify the responsible actor for model development, integration, local validation, routine monitoring, incident review, user training, and authority to suspend use, together with handoff points between actors, reporting obligations, and escalation routes when clinically meaningful deterioration or harm is detected. In this way, accountability is translated from an abstract governance principle into a deployable institutional requirement.

### AI-Mediated Care Relationships

Clinical AI may increasingly mediate communication, guidance, and continuity across patient journeys, particularly in navigation, triage, symptom support, and longitudinal follow-up [16,17]. In these settings, governance concerns extend beyond safety and performance to the quality of AI-mediated relationships, including trust, continuity, dependence, and the conditions under which patients can access meaningful human interaction. Existing governance frameworks remain limited in how they address relational quality, continuity safeguards, or the risk that AI-mediated support may alter help-seeking, disclosure, or perceptions of professional care. Governance should therefore address not only what these systems do but also how they reshape communication and care relationships over time. Minimum safeguards may include clear disclosure of AI mediation, defined routes to human escalation, continuity protections for higher-risk or vulnerable patients, and review of relational impacts in longitudinal deployments.

## Shifting Roles Across the Clinical AI Governance Landscape

### Overview

Futures methodology attends not only to technological change but also to the shifting human and institutional roles that accompany system transition. As clinical AI evolves across horizons, the roles of clinicians, patients, developers, operators, and regulators will also change, with direct governance consequences. Frameworks that fail to anticipate these shifts risk creating accountability gaps, competency mismatches, and consent structures that no longer reflect the actual distribution of agency in AI-enabled care.

### Clinicians: From Practitioners to AI Supervisors

In H1 settings, the clinician’s role remains broadly recognizable: AI provides a recommendation, and the clinician accepts, modifies, or overrides it using independent professional judgment [2,12]. As AI outputs become more numerous, complex, and time-sensitive, however, the conditions for meaningful oversight may erode, as earlier work on alert fatigue in clinical decision support suggests [13,14,38]. In H2 and beyond, the clinician’s role may shift from direct execution of care toward supervision of AI-enabled processes, requiring competencies not central to traditional clinical practice, including interpretation of outputs in light of known limitations, detection of behavioral drift, and communication of uncertainty to patients [32,39]. Current professional education and credentialing structures are not yet aligned with this shift [40]. Governance should therefore recognize AI oversight competency as a core component of professional preparation, clinical privileging, and continuing education.

### Patients: From Recipients to Conavigators

Patients have received less attention than clinicians or regulators in clinical AI governance, and in many current deployments, they remain passive recipients of AI-mediated recommendations, often without clear disclosure of AI involvement [6,25,41]. Informed consent frameworks also remain underdeveloped, with no settled standard for what patients should be told about AI’s role, limitations, or variable performance across populations [16,42]. In longer-horizon settings, patients may interact directly and longitudinally with AI-enabled navigation, triage, or communication tools, raising governance questions about continuity, therapeutic alliance, the right to meaningful human contact, and the boundary between delegation and substitution [43]. Patient rights in AI-mediated care should therefore be treated as a core governance domain rather than a narrow extension of disclosure requirements [18].

### Developers and Operators: From Innovators to Governors

Clinical AI governance increasingly depends on the actions of developers and deployers well beyond model creation [1,8]. The EU AI Act’s distinction between providers and deployers reflects this shift by assigning obligations to both those who place AI systems on the market and those who implement them in operational settings [4,7]. In health care, governance must therefore extend across the deployment life cycle and not stop at technical development alone [10,15]. Developers should disclose key features of training data provenance, known performance limitations, and relevant subgroup performance where feasible [25,29]. Deployers and operating institutions should document deployment context, local validation, monitoring procedures, and escalation mechanisms for clinically meaningful deterioration or adverse events [8,37]. The governance principle is clear: stewardship responsibilities should follow the system through its life cycle rather than end at market entry [15,21].

### Regulators: From Gatekeepers to Adaptive Stewards

The traditional regulatory model, in which regulators primarily act as gatekeepers evaluating products against predefined standards [3,4], is increasingly insufficient for systems whose behavior, use context, and organizational effects may change after deployment [10,24]. A futures-oriented approach suggests that regulators will also need adaptive stewardship capacities, including monitoring population-level performance, updating expectations in response to postdeployment evidence, and convening multistakeholder processes when new governance issues emerge [21,22]. International coordination will be especially important for systems operating across jurisdictions [5], and bodies such as the International Medical Device Regulators Forum may provide useful mechanisms for convergence in classification, postmarket monitoring, and life cycle oversight of AI-enabled medical technologies [44].

## Scenario Planning for Clinical AI Regulatory Design

Scenario planning offers a useful method for exploring divergent futures without requiring deterministic prediction [45]. It enables governance designers to examine how technological, institutional, and regulatory conditions may interact and to stress-test policies against a small number of coherent but meaningfully distinct scenarios without assuming a single expected trajectory [19,21].

### Scenario Structure and Governance Logic

In clinical AI governance, scenario planning is especially valuable because uncertainty arises not only from the pace of technological development but also from the coherence and adaptability of regulatory response [21,22]. FF-CAIG uses 2 dimensions: the pace of clinical AI capability development, from incremental to rapid and broadly integrated, and the coherence of regulatory response, from fragmented and reactive to coordinated and anticipatory [45]. On this basis, we focus on 3 analytically salient futures: governed acceleration, fragmented proliferation, and regulatory retrenchment (Table 3). The purpose is not to predict which future will occur, but to identify governance choices that remain robust across plausible trajectories [45].

**Table 3:** Scenario matrix for clinical artificial intelligence (AI) regulatory futures.

Domain	Scenario A: governed acceleration	Scenario B: fragmented proliferation	Scenario C: regulatory retrenchment
Regulatory environment	Adaptive and increasingly harmonized frameworks; stronger life cycle oversight and cross-jurisdictional alignment	Patchwork regulation; inconsistent enforcement; uneven institutional governance capacity	Postincident restriction, higher approval thresholds, and defensive compliance culture
Clinician role	AI oversight embedded in training, credentialing, and practice standards	Variable expectations across specialties, settings, and resource levels	Greater risk aversion; reduced willingness to rely on AI; increased documentation and liability burden
Equity outcomes	Equity integrated into validation, procurement, and postdeployment monitoring; routine subgroup reporting	Benefits and monitoring concentrated in well-resourced institutions; disparities widen	Formal equity attention may increase, but access gaps and structural inequities persist
Innovation trajectory	Trust-supported growth under clearer rules and accountability	Rapid but uneven expansion outpaces governance and standardization	Slower innovation; resources shift toward compliance, legal defense, and risk containment

### Scenario-Based Stress Testing for Governance Design

#### Scenario A: Governed Acceleration

In this pathway, innovation is matched by adaptive regulatory frameworks, stronger postdeployment monitoring, and greater alignment across institutions and jurisdictions [21,22]. Public trust is reinforced through transparency, accountability, and meaningful inclusion of clinician and patient perspectives [18,25]. Equity is treated as a core implementation requirement rather than a secondary concern [29,31].

#### Scenario B: Fragmented Proliferation

In this pathway, clinical AI capabilities expand while governance remains uneven across jurisdictions, organizations, and specialties [5,15]. Adoption proceeds rapidly but inconsistently, with safety protections, clinician support, and postmarket oversight varying by institutional resources and vendor practices [8]. The likely result is a widening disparity in who benefits from clinical AI and under what conditions [31,33].

#### Scenario C: Regulatory Retrenchment

In this pathway, high-profile AI-related patient safety failures trigger more restrictive control and intensify approval burdens [3]. Although politically understandable, this response may inhibit beneficial innovation without correcting the structural weaknesses that contributed to the precipitating harm [10,24].

Within FF-CAIG, scenario planning functions as a structured stress-testing tool for regulatory proposals, institutional strategies, and professional standards. Life cycle monitoring, clearer accountability allocation, clinician oversight competencies, and routine equity surveillance are likely to remain valuable across all 3 scenarios [2,8,15]. By contrast, governance strategies that assume stable technological trajectories or uniformly responsible deployment are more fragile under fragmented or crisis-driven futures [10,21].

## CLA and the Deep Structures of Clinical AI Governance

### Overview

CLA offers a useful framework for examining how visible governance problems are sustained by deeper structural, cultural, and narrative conditions [46]. It distinguishes 4 layers of analysis: visible problems, systemic drivers, worldview assumptions, and underlying myths and metaphors [46]. In clinical AI governance, issues such as algorithmic bias, accountability gaps, and opaque decision-making may therefore reflect not only technical failures but also deeper assumptions about clinical knowledge, epistemic authority, and the relationship between algorithmic efficiency and human care [29,42].

### Applying CLA to Clinical AI Governance

At the litany level, the visible concern is an AI system that produces biased recommendations, underperforms in certain patient groups, or fails silently after deployment [29,30]. At the systemic level, these failures may be reinforced by incentive structures that privilege aggregate performance over subgroup equity [31], regulatory processes that rely heavily on developer-provided evidence [10], and procurement practices that prioritize efficiency over long-term oversight capacity [1,8]. At the worldview level, governance may be shaped by techno-optimism that equates AI adoption with progress and frames skepticism as resistance to innovation [39]. At the myth and metaphor level, the image of AI as a precision instrument may obscure the extent to which algorithmic outputs are shaped by assumptions embedded in design and deployment [42].

To illustrate how these 4 layers operate together in practice, consider an AI-enabled triage tool that underescalates symptoms reported by a particular subgroup of patients [29]. CLA would examine not only this visible failure but also the systemic factors that produced it, such as underrepresentation in training data, limited subgroup testing, procurement criteria emphasizing throughput, or weak postdeployment monitoring. The analysis would also surface worldview assumptions that AI-enabled triage is inherently efficient and objective, as well as deeper metaphors of AI as a neutral gatekeeper. The resulting governance response would extend beyond model correction to include procurement standards, subgroup surveillance, escalation pathways, access to human review, and examination of the institutional assumptions that allowed the risk to remain underrecognized.

### From Causal Layers to Governance Interventions

The governance value of CLA lies in changing the object of intervention [46,47]. If the problem is framed only at the litany level, the likely response is to add bias audits, incident reporting, or postdeployment monitoring [29,31]. If CLA reveals that the problem is also systemic, governance must extend to procurement criteria, local validation requirements, committee composition, and allocation of monitoring responsibility [8,15,37]. If the problem is partly rooted in worldview assumptions, such as equating AI adoption with modernization or treating skepticism as resistance to innovation, decision-makers may need to revise the criteria by which clinical AI is approved, funded, or scaled [39,47]. If deeper myths and metaphors frame AI as a neutral precision instrument instead of a sociotechnical actor, governance may need to include explicit review of relational effects, epistemic dependency, and institutional power asymmetries [42,47,48]. Within FF-CAIG, CLA therefore helps regulators and health systems decide not only how to govern AI but also which level of governance intervention is required and why [46].

## Applying FF-CAIG: An Operational Workflow

FF-CAIG’s analytic components yield governance value only when they inform concrete deployment decisions. We therefore propose a 5-step workflow, summarized in Table 4, to translate the framework into a structured review process for AI procurement, deployment authorization, postdeployment reassessment, and regulatory policy design.

In step 1, reviewers characterize the system under review, including its clinical function, data sources, update mechanism, autonomy level, deployment setting, patient-facing role, anticipated clinical consequences, and responsible actors. In step 2, the system is assigned to 1 or more horizons using 4 operational criteria: prevalence in routine care, level of real-time human oversight, fit with existing governance instruments, and observability of associated risks. This classification clarifies which risks are already governable, which require transitional adaptation, and which may fall outside current oversight instruments. In step 3, reviewers apply FF-CAIG’s analytic components by identifying implicated risk domains, stress-testing governance responses across plausible scenarios, examining deeper structural and cultural assumptions through CLA, and mapping accountability across actors. In step 4, reviewers consolidate the preceding analyses into a governance review record that specifies monitoring obligations, accountability allocation, disclosure and oversight requirements, equity surveillance expectations, and conditions for modification or suspension. In step 5, reviewers specify minimum actions scaled by horizon: a baseline for H1 systems centered on validation, monitoring, escalation, human fallback, and suspension authority, with additional H2 and H3 safeguards for life cycle monitoring, autonomy limits, patient-facing disclosure, access to human review, and reassessment after material change.

**Table 4:** Operational workflow for applying the futures framework for clinical artificial intelligence governance.

Workflow step	Core focus	Governance output	Minimum action
1. Characterize the system	Clinical function, data sources, update mechanism, autonomy level, deployment context, patient-facing role, and responsible actors	System profile and deployment context	Document technical, organizational, and relational system characteristics
2. Assign horizon classification	Prevalence in routine care, real-time human oversight, fit with existing governance instruments, and risk observability	H1^a^, H2, H3, or multihorizon classification	Assign horizons and record the rationale for horizon placement
3. Apply analytic components	Risk domains, scenario stress-testing, causal layered analysis, and accountability across actors and handoffs	Risk-domain map, scenario findings, causal layered analysis findings, and accountability-chain map	Assess the deployment across risk domains, scenarios, causal layers, and accountability chains
4. Define governance outputs	Monitoring, accountability, disclosure, equity surveillance, and conditions for modification or suspension	Consolidated governance review record	Specify monitoring indicators, responsibility allocation, disclosure requirements, equity surveillance, and pause or suspension criteria
5. Set minimum actions	Horizon-scaled governance floor for deployment or continued use	Deployment authorization, conditional approval, modification requirement, or nondeployment decision	Require H1 baseline safeguards; add life cycle monitoring, autonomy limits, disclosure, human review access, and reassessment for H2-H3 systems

^a^H: horizon.

## Applying FF-CAIG to Representative Clinical AI Deployments

### Overview

The following 3 representative deployment patterns illustrate how this workflow can be applied across the horizon continuum. These examples are literature-grounded analytic exemplars that also reflect emerging institutional practice; they are not empirical validation exercises or single-site implementation studies. They demonstrate how FF-CAIG can be applied across different governance horizons, risk constellations, and deployment architectures.

### Electronic Health Record–Embedded Foundation Model Documentation Assistant (H1)

A large academic medical center deploys a vendor-supplied ambient documentation assistant integrated into the electronic health record. FF-CAIG places this use case primarily in H1, while recognizing emerging H2 risks, as deployment expands across specialties and patient populations. The main governance concerns are model drift and distributed liability across the vendor, electronic health record integrator, deploying institution, and end users [8,15,17]. The governance response is therefore focused on regular disaggregated performance reporting, a named institutional lead for monitoring and escalation, and clinician onboarding on system limitations and override conditions. Scenario stress-testing identifies a key vulnerability: vendor-initiated model updates may alter subgroup performance without timely local detection [26,27]. The resulting H1 governance requirement is a deployment authorization standard that ties continued use to defined monitoring, reporting, and accountability obligations.

### Patient-Facing Longitudinal Triage and Communication Tool (H2)

A regional health system introduces an AI-enabled patient navigation tool for longitudinal symptom monitoring, triage, and follow-up messaging between clinic visits. FF-CAIG places this deployment in H2 because the system interacts directly with patients over time while exercising increasing communicative and triage autonomy without routine clinician review. The key risk domains are agentic autonomy, AI-mediated care relationships, and opacity about AI involvement [16,43,47]. Governance priorities, therefore, include defined escalation thresholds, plain-language disclosure of AI use, access to human review on request, and exclusion criteria for patient groups requiring additional safeguards [16]. Stress-testing of expansion into outpatient follow-up and longitudinal care further highlights the need for explicit rules governing use in patients with impaired decision-making capacity.

### Autonomous AI-Based Screening Agent (H3)

A health system considers deploying an AI agent that autonomously reviews flagged imaging studies, generates preliminary reports, and triages cases for expedited review before radiologist assessment. FF-CAIG classifies this as H3 because clinically consequential action occurs with limited real-time human oversight [36]. The main governance concerns are agentic autonomy, distributed liability, and equity risk arising from uneven performance across subpopulations [29,31]. The governance response, therefore, requires predefined limits on autonomous action, thresholds for suspension or human fallback, and clear institutional responsibility for monitoring and intervention [8,15]. Stress-testing across affiliated community hospitals shows that validation in an academic center alone is insufficient. Deployment should therefore require site-specific validation, subgroup-based suspension thresholds, and clearly assigned authority to halt use when safety or equity criteria are breached [29,31,36].

## Cross-Horizon Governance Priorities

### Overview

Drawing on the 3H model, the emerging risk taxonomy, scenario planning, and CLA, FF-CAIG identifies horizon-scaled governance priorities for near-term, transitional, and longer-term clinical AI environments. These priorities do not constitute separate risk categories; rather, they translate the preceding analysis into governance emphases that vary according to system maturity, autonomy, clinical consequences, institutional capacity, and regulatory uncertainty.

### Short-Term Priorities (H1)

Near-term priorities should strengthen the governance foundations for currently deployed high-risk clinical AI systems by making them more auditable, locally accountable, and equity-aware. For high-impact systems, the core requirements include predeployment validation proportionate to clinical risk, subgroup-disaggregated performance evaluation, documentation of intended use and known limitations, and clear assignment of responsibility for monitoring, escalation, and suspension [9,17,29,31,35,37]. In parallel, clinician education, continuing professional development, and, where appropriate, credentialing expectations should begin to align with emerging AI oversight roles [40]. These measures address risks already visible in current clinical AI deployments while creating the baseline infrastructure needed for later life cycle oversight.

### Medium-Term Priorities (H2)

Transitional priorities should focus on systems that are iteratively updated, workflow-embedded, foundation model–based, or partially delegated [32,34]. In this horizon, governance should move from one-time validation toward life cycle review, with explicit thresholds for clinically meaningful performance change, reassessment after material updates, and standards for meaningful human oversight [9,26,27,36,39]. Patient-facing and workflow-integrated systems also require proportionate safeguards when AI materially shapes triage, communication, or care coordination, including disclosure, escalation pathways, and review mechanisms appropriate to clinical risk [16,42].

### Long-Term Priorities (H3)

Longer-term priorities should address clinical AI systems that function as persistent, autonomous, or AI-mediated care infrastructures. Governance in this horizon should define the conditions under which autonomous or low-human-intervention clinical actions are permissible, how accountability is allocated across distributed systems, and how patients are protected when AI mediates access, communication, or continuity of care [36,39,43,47]. These priorities require stronger institutional and cross-jurisdictional coordination because the relevant risks may exceed the capacity of individual organizations or conventional product-centered oversight [44]. Across horizons, the central governance task is not simply to regulate more AI, but to align governance intensity, accountability structures, and relational safeguards with changing forms of clinical AI capability and deployment.

These priorities are largely convergent, with recommendations already present in the clinical AI governance literature, including life cycle oversight, equity auditing, distributed accountability, and human oversight standards. They should also be interpreted alongside evolving cross-sector and international AI governance instruments, including the EU AI Act [49], the National Institute of Standards and Technology AI Risk Management Framework and its generative AI profile [50,51], and International Organization for Standardization/International Electrotechnical Commission standards on AI management systems and AI risk management [52,53]. FF-CAIG’s contribution is therefore not to introduce these priorities as isolated new requirements, but to show how their relative importance shifts across temporal horizons and divergent regulatory futures. By linking governance priorities to emerging risks, shifting roles, regulatory uncertainty, and deeper sociotechnical assumptions, FF-CAIG situates established governance commitments within a single anticipatory framework and clarifies when existing safeguards may remain sufficient, when they require adaptation, and when new forms of governance may become necessary.

## Limitations

Several limitations and tensions in anticipatory governance warrant acknowledgment. First, front-loading governance requirements may increase compliance burden, especially for smaller developers, community hospitals, and health systems with limited governance infrastructure. Measures such as site-specific validation, disaggregated performance reporting, and accountability-chain documentation are more easily absorbed by large academic centers and well-resourced technology firms than by safety-net institutions or early-stage developers [8,15,31]. If these requirements become de facto barriers to entry, anticipatory governance may contribute to regulatory consolidation without producing broader safety improvement. FF-CAIG does not resolve this tension and therefore requires jurisdictional and institutional adaptation to avoid becoming an instrument of exclusion.

Second, anticipatory governance carries a risk of overdocumentation and procedural burden. Governance mechanisms designed for H2 and H3 conditions may be premature or disproportionate when applied to current H1 deployments, and prospective planning requirements may divert institutional capacity from direct patient care or quality improvement. A tiered approach that scales governance intensity to the autonomy, scope, and clinical consequences of the AI system may reduce this risk, although operationalizing such proportionality remains an open challenge.

Third, the feasibility of FF-CAIG will vary across health systems and regulatory contexts. The framework draws primarily on regulatory developments in the United States and European Union [4,7,9]. Consequently, some recommendations may be difficult to implement in jurisdictions with less developed AI governance infrastructure, limited health informatics capacity, or different legal frameworks for medical device oversight. Equity in access to anticipatory governance tools is, therefore, itself a governance challenge that the framework does not fully resolve.

Fourth, the deployment patterns used to illustrate FF-CAIG are representative analytic constructs, not empirical case studies. They are intended to demonstrate the framework’s interpretive and anticipatory logic across horizons, not to validate its real-world effectiveness. Relatedly, the cross-horizon priorities are derived analytically from the convergence of the framework’s temporal and scenario analyses; they have not been formally validated or systematically compared against alternative governance approaches. Future work should validate and refine FF-CAIG and its proposed cross-horizon priorities through modified Delphi consensus, retrospective mapping of documented clinical AI incidents or governance reviews, and prospective pilot-testing within institutional AI governance committees to assess horizon classification agreement, risk identification completeness, accountability clarity, decision documentation, and reviewer-reported usefulness.

Fifth, FF-CAIG was not developed through primary expert consultation, Delphi elicitation, or structured stakeholder interviews, and therefore does not claim direct empirical grounding in the views of regulators, clinicians, patients, developers, or health-system leaders. Stakeholder perspectives informed the framework indirectly through the peer-reviewed studies, regulatory and policy documents, position statements, and international guidance included in the targeted narrative review. However, documentary representation is not a substitute for direct multistakeholder input, particularly for patient perspectives, which remain underrepresented in the clinical AI governance literature. Future Delphi-based validation should therefore examine whether FF-CAIG captures concerns that documentary sources may underweight or omit.

## Conclusions

Clinical AI governance is entering a consequential period of transition. Decisions made over the next several years will shape whether clinical AI mitigates or amplifies existing inequities; whether patients retain meaningful rights to disclosure, explanation, and access to human review in AI-mediated care; and whether health systems develop the oversight capacity needed for adaptive and increasingly complex AI systems. FF-CAIG offers a structured approach for moving governance from a predominantly reactive model toward a more anticipatory one. It can help regulators, health system leaders, developers, and researchers identify cross-horizon governance gaps, stress-test governance strategies against divergent futures, and align oversight with the changing capabilities and consequences of clinical AI. Clinical AI governance should therefore be understood not simply as a technical regulatory task, but as a sociotechnical and institutional challenge requiring prospective, systems-oriented, and stakeholder-engaged approaches. Futures methodologies cannot eliminate uncertainty, but they can support more disciplined preparedness before emerging risks fully crystallize.

## Abbreviations

3H: three horizons
AI: artificial intelligence
CLA: causal layered analysis
FDA: US Food and Drug Administration
FF-CAIG: futures framework for clinical artificial intelligence governance

## Data Availability

Data sharing is not applicable to this paper because no datasets were generated or analyzed during this study.
